# Individual differences in age-related neurocognitive outcomes: within-subject assessment of memory for odors

**DOI:** 10.3389/fnagi.2023.1238444

**Published:** 2023-09-28

**Authors:** Audrey E. Branch, Lucas R. Glover, Michela Gallagher

**Affiliations:** ^1^Department of Psychological and Brain Sciences, Johns Hopkins University, Baltimore, MD, United States; ^2^Solomon H. Snyder Department of Neuroscience, Johns Hopkins School of Medicine, Baltimore, MD, United States; ^3^Johns Hopkins Kavli Neuroscience Discovery Institute, Johns Hopkins University, Baltimore, MD, United States

**Keywords:** cognitive aging, recognition memory, spatial memory, medial temporal lobe, odor recognition memory

## Abstract

Cognitive decline is a common feature of aging, particularly in memory domains supported by the medial temporal lobe (MTL). The ability to identify intervention strategies to treat or prevent this decline is challenging due to substantial variability between adults in terms of age of onset, rate and severity of decline, and many factors that could influence cognitive reserve. These factors can be somewhat mitigated by use of within-subject designs. Aged outbred Long-Evans rats have proven useful for identifying translationally relevant substrates contributing to age-related decline in MTL-dependent memory. In this population, some animals show reliable impairment on MTL-dependent tasks while others perform within the range of young adult rats. However, currently there are relatively few within-subject behavior protocols for assessing MTL function over time, and most require extensive training and appetitive motivation for associative learning. In the current study, we aimed to test whether water maze learning impairments in aged Long-Evans rats would be predictive of delayed recognition memory impairments and whether these odor memory impairments would be stable within subjects over multiple rounds of testing.

## 1. Introduction

Age-related cognitive decline in otherwise healthy individuals has a high societal cost but presents a considerable challenge for study. Not all aging individuals exhibit neurocognitive decline and those that do have distinctive profiles of cognitive complaints and trajectories of decline. Given these individual differences in aging, efforts to identify the underlying neurobiological basis of decline are likely to be more successful when behavioral and biological markers are assessed in the same subject. While this heterogeneity is well documented in human literature and has been shown to be a conserved feature of aging across species, there is a relative deficit in the number of cognitive testing paradigms for assessing this phenomenon in animal models. Cognitive testing paradigms which accurately assess individual cognitive differences in animals and stratify according to cognitive ability represent a powerful approach to identifying underlying neurobiological features contributing to age-related cognitive decline and for assessing potential treatments (Baxter and Gallagher, 1996; Logan et al., 2023).

One very common complaint for individuals experiencing age-related cognitive decline is the ability to form and retrieve episodic memories, suggesting dysfunction in the medial temporal lobe (MTL) memory system. The MTL plays an essential role in the formation of episodic memories and research focused on episodic memory in humans, monkeys, and rodents has made strides in understanding the contribution of MTL cortical areas and the hippocampal formation across species (Mishkin, 1982; Fortin et al., 2004; Guderian et al., 2011; Kim et al., 2020; Cooper et al., 2022). Aging is commonly associated with a diminution of episodic memory capacity; however, the rate and severity of this decline varies widely across individuals. Maintenance of memory function, referred to as successful aging, can occur in some aged humans while others in older cohorts are impaired (Davis et al., 2003; Nyberg et al., 2012; Salami et al., 2018). The observation that non-human primates (Rapp et al., 1997) and aged outbred rodents (Gallagher and Burwell, 1989; Gallagher et al., 1993) also exhibit such individual differences in memory decline provides an opportunity to model this phenomenon for studies of underlying mechanisms in experimental laboratory research.

In aged rodents, spatial memory assessments, such as the Morris water maze, are commonly used to identify MTL-dependent memory impairments. In a well-characterized population of outbred Long Evans rats, age-dependent cognitive performance includes both learning impairments (aged-impaired, AI) and preserved cognitive function on par with young adults (aged-unimpaired, AU) (Gallagher et al., 1993). When pre-screened for spatial memory performance in the Morris water maze, learning scores for these animals have been shown to be stable over time (Gallagher and Burwell, 1989; Colombo et al., 1997) and predictive of performance on subsequent learning tasks that depend on the functional integrity of the medial temporal lobe, including the hippocampus (Koh et al., 2010, 2013, 2020). Importantly, individual differences in MWM performance in aged Long-Evans rats has been shown to be highly correlated with differences in neurobiological markers of circuit integrity in the MTL (Colombo et al., 1997; Smith et al., 2000; Lee et al., 2005; Haberman et al., 2011; Stranahan et al., 2011; Tran et al., 2018).

Here we use a spontaneous novel odor recognition protocol to further assess the reliability of aged outbred Long-Evans rats as memory-impaired and memory-unimpaired relative to young adults. Specifically, both young adult and aged animals characterized for spatial memory were given short- and long-term recognition memory assessments using olfactory odor sets during three distinct experiences across unique contexts. We found that both young adult and aged animals displayed good recognition memory for odors over a short-term delay, but only those aged animals with poor spatial learning scores were impaired after a long-term 24-h delay. This impairment exhibited within-subject reliability for individual differences in neurocognitive aging over repetitions with new odorant cues, and thus may serve as a basis for multiple rounds of testing in future studies of interventions.

## 2. Methods

### 2.1. Animals

All procedures were approved by the Institutional Animal Care and Use Committees in accordance with the National Institutes of Health directive. Male Long-Evans rats were obtained at 8–9 months of age from Charles River Laboratories (Raleigh, NC). They were housed in a vivarium at Johns Hopkins University until they were 24–26 months old for assessment of aged rats. Young adult rats were obtained from Charles River and were housed in the same vivarium. Rats were individually housed in cages containing corncob bedding and constant ventilation. The vivarium was 25°C and on a 12-h light/dark cycle (lights on at 7 A.M.). Water and food were provided *ad lib.* Rats were continuously monitored for health. Pathogen-free status and necropsies were performed at the time of sacrifice. Rats that showed impaired health or disabilities that could impact behavioral performance (e.g., poor eyesight, clinical evidence of renal impairment, pituitary or other tumors) were excluded from the study. All procedures were approved by the Johns Hopkins University Institutional Animal Care and Use Committee in accordance with the National Institutes of Health directive.

### 2.2. Background behavioral characterization

**Figure 1:**
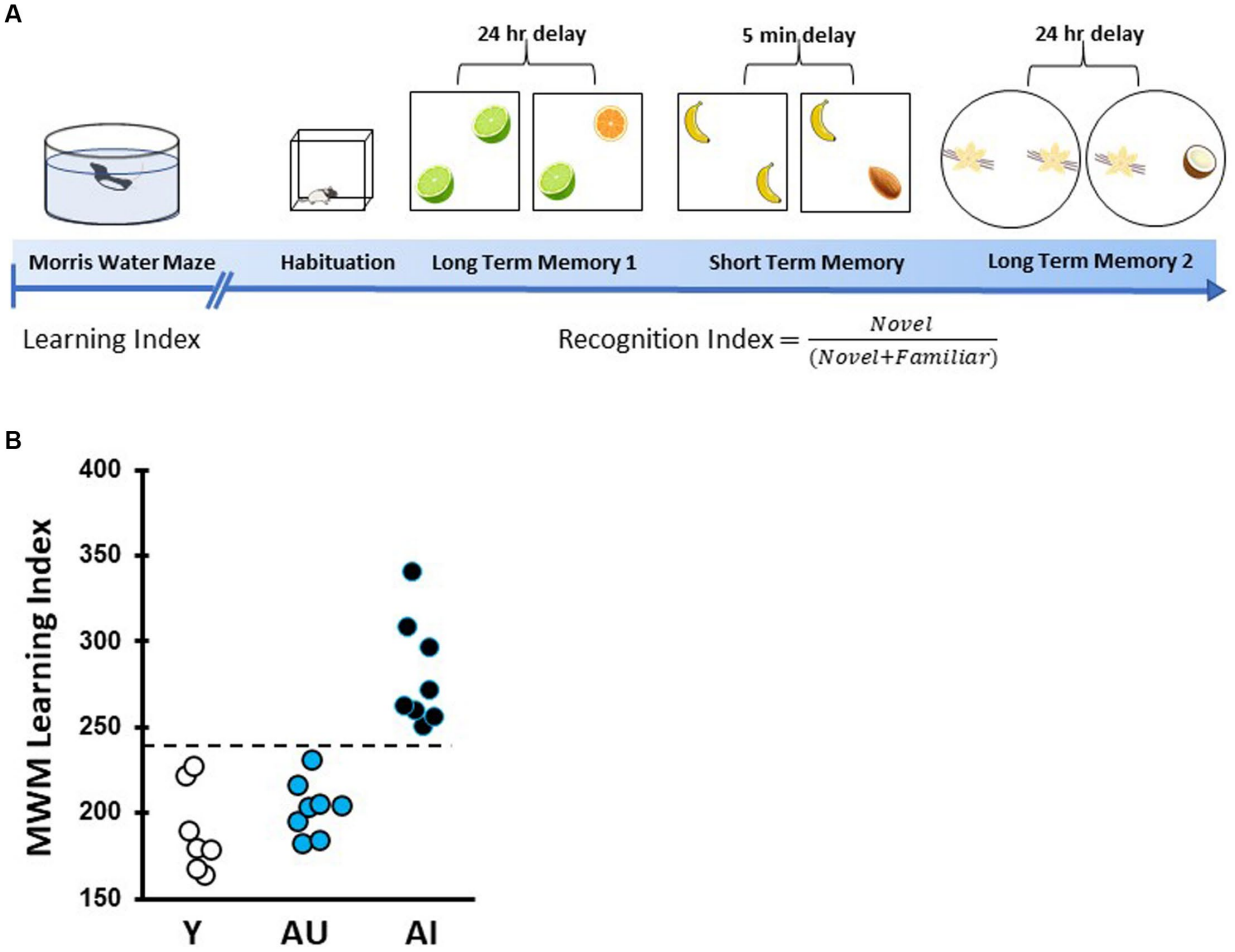
Timeline of spatial learning characterization and odor recognition behavioral assessment. **(A)** Rats were first characterized for intact or impaired hippocampal-dependent learning ability via the Morris water maze (MWM). This was followed by handling and open field habituation prior to odor recognition tests. Rats were then given three novel odor sets for recognition memory tests with a 24-h (long-term memory) or 5-min (short-term memory) delays. **(B)** MWM learning index scores for each animal were derived from proximity measures during four probe trials interpolated throughout training as in Gallagher et al. (1993), with lower scores indicating better performance. Aged unimpaired animals perform within the range of young animals (<240) while age impaired (AI) perform more poorly (>240).

Young adult (8–9 months) and aged rats (24 months) were tested in an assessment of hippocampal function prior to odor recognition memory behavioral tests. The background behavioral assessment used a well-established Morris water maze protocol as described in detail elsewhere (Gallagher et al., 1993; Tomás Pereira and Burwell, 2015). This protocol was designed to tax memory in the task with sparse training (3 trials per day) at 24-h intervals. Rats were trained for 8 days (3 trials per day) to locate a camouflaged escape platform that remained at the same location throughout training in a water maze surrounded by curtains with fixed cues. Every sixth trial consisted of a probe trial (no escape platform for the first 30s of the trial) that served to assess the development of a spatially localized search. Learning Index (LI) scores were derived from each rat’s proximity to the platform during the four probe trials. The proximity measure was obtained by sampling the position of the animal in the maze (10 times per second) to provide a record of its distance from the escape platform in 1-s averages. The learning index is the sum of weighted proximity scores obtained during probe trials, with low scores reflecting a more accurate search and indicating better retention of the platform location. A learning index cutoff was used to identify aged rats as Aged Unimpaired (“AU”) or Aged Impaired (“AI”). The cut off value was an index score of 240, with higher scores representing worse performance and reflecting scores that fall inside or outside the normative range collected from young adult Long-Evans rats over many years. Cue training was used to assess the sensori-motor and motivational status of the rats. Only rats with successful cue training performance were included in the present study. After behavioral characterization and cue training, a total of 23 rats, including Y (*n* = 7), AU (*n* = 8), and AI (*n* = 8) rats were selected (Figure 1B). One AI died before completing short-term delay test, and one AU did not meet minimum exploration criteria for the first long-term delay task.

### 2.3. Olfactory recognition memory test

Six weeks after MWM characterization, odor recognition memory was assessed over a 15-day protocol using odor sets in three within-subject assessments. A square arena (70 × 70 × 50 cm) was used for long- and short-term memory testing and a circular arena (diameter = 76 cm) was used for a second long-term memory test to provide a distinctly different context. Both arenas were grey and were surrounded by a black curtain during sample and test phases. The bottom of each arena was covered with ~2 cm of corncob bedding which was refreshed between counterbalanced groups, each consisting of AU, AI, and Y rats (Antunes and Biala, 2012). Odors were made fresh each day with 200 μL of odor liquid extract (McCormick, Hunt Valley, MD) diluted in 1 mL of distilled water and placed on a gauze pad inside a scintillation vial which was fastened to the arena floor with Velcro. In the square and the circular arenas, odor vials were placed 10 cm from the walls. Odor concentration and identity was based on previous work showing that young adult and aged rats show no preference for or aversion to these odorants under these conditions (Weiler et al, 2021). The sequence of behavioral tasks is outlined in Figure 1A.

In the preliminary habituation and acclimation phase, rats were handled for at least 5 min per day for 3 days and then allowed to freely explore the test arena for 10 min a day for 5 days. Following this, animals were given a sampling phase in which two vials containing identical odors were introduced into the arena and rats were allowed to explore for 10 min. After a 24-h delay, the animals were placed in the square arena with two vials, one containing the odorant present on the sampling phase and the other containing a novel odor (Long-term Memory 1, LTM1) (Weiler et al., 2021). After a 1-week delay, this was followed by a Short-term Memory (STM) test in the square arena in which animals were given a sampling phase with two identical odors followed by the test phase with one novel and one familiar odor, with a 5-min delay between test and sample phases. Finally, after a 1-week delay animals were given a second Long-term Memory test (LTM2) in a circular arena with a new set of odors. All animals were given the same pair of odorants for each test, with the identity of novel and familiar odors pseudo-counterbalanced across animals (LTM1: lime and orange, STM: banana and almond, LTM2: vanilla and coconut). For all sample and test phases, animals were allowed to freely explore for 10 min. All behavior occurred under red light with overhead lights off and with a white-noise generator on. All phases were digitally recorded for offline scoring between three experimenters.

For scoring during the sample and testing phases, exploration time was defined as the time the rat’s snout was either in or directly above the vial. A Recognition Index (RI) score [(novel)/(novel + familiar)] was calculated, as previously described for object and odor recognition (Ennaceur and Delacour, 1988; Weiler et al., 2021). An RI of 0.5 represents no odor exploration preference, reflecting a lack of recognition memory of the sample phase odor. All scoring was performed by raters who were blind to experimental conditions and scoring was confirmed across three different raters (Supplementary Figure S1). Any rat that explored odor vials for less than 5 s during the sampling or testing phase was excluded from further analysis.

### 2.4. Statistics

The Kolmogorov–Smirnov test was used to test for normal distribution of the data, and Bartlett’s test was used to test for equality of variances for all variables. Memory retention for sample phase odors was investigated using one-sample t-tests, comparing RI scores to 0.5 (i.e., comparing investigation preference for the novel odor to chance). To assess differences in recognition index memory in each task, one-way ANOVAs were performed with group as the independent factor with Tukey’s HSD post-hoc tests and Cohen’s d statistics where appropriate. Correlations between learning index and recognition index were determined with Spearman rank correlation coefficient and correlations between repeated recognition index scores were determined with Pearson correlation coefficient. Statistical tests were performed using GraphPad Prism version 10.0.0 for Windows (GraphPad Software, Boston, Massachusetts United States, www.graphpad.com). Full results of statistical tests not reported in the text are reported in Supplementary Table S1.

## 3. Results

### 3.1. Age-related spatial learning deficits parallel long-term odor recognition memory impairments

As illustrated schematically in Figure 1A, all rats used in this study were first assessed for individual differences in spatial learning followed by three tests of odor recognition memory at different delays. Spatial memory performance was assessed using the hidden platform water maze protocol developed in this study population and optimized for sensitivity to detect individual differences in aging apart from confounds due to physical disability or pathological conditions (Gallagher et al., 1993). Higher learning index (LI) scores signify worse performance by reflecting search at a greater distance from the escape location during memory probe tests. Figure 1B shows the learning index distribution derived from task performance over the four probe trials for young adult control (*n* = 7) and all aged rats (*n* = 16) used in this study. A repeated-measures, two-way ANOVA confirmed rats improved with training block (*F* (4, 80) = 187.8, *p* < 0.001) with the last day showing differences between age groups.

As found in this study population and in the subset of animals used here, AU rats’ performance is on par with Y rats while AI rats fell outside the range of normative distribution of young adult rats (LI scores ≤240 and > 240 were classified as AU and AI, respectively). An overall one-way ANOVA of learning index demonstrated significant differences across age (*F* (2, 20) = 29.24, *p* < 0.0001). Y and AU groups differed significantly from AI rats (both groups *p* < 0.0001) but LI scores were not different for AU and Y rats (*p =* 0.98).

**Figure 2:**
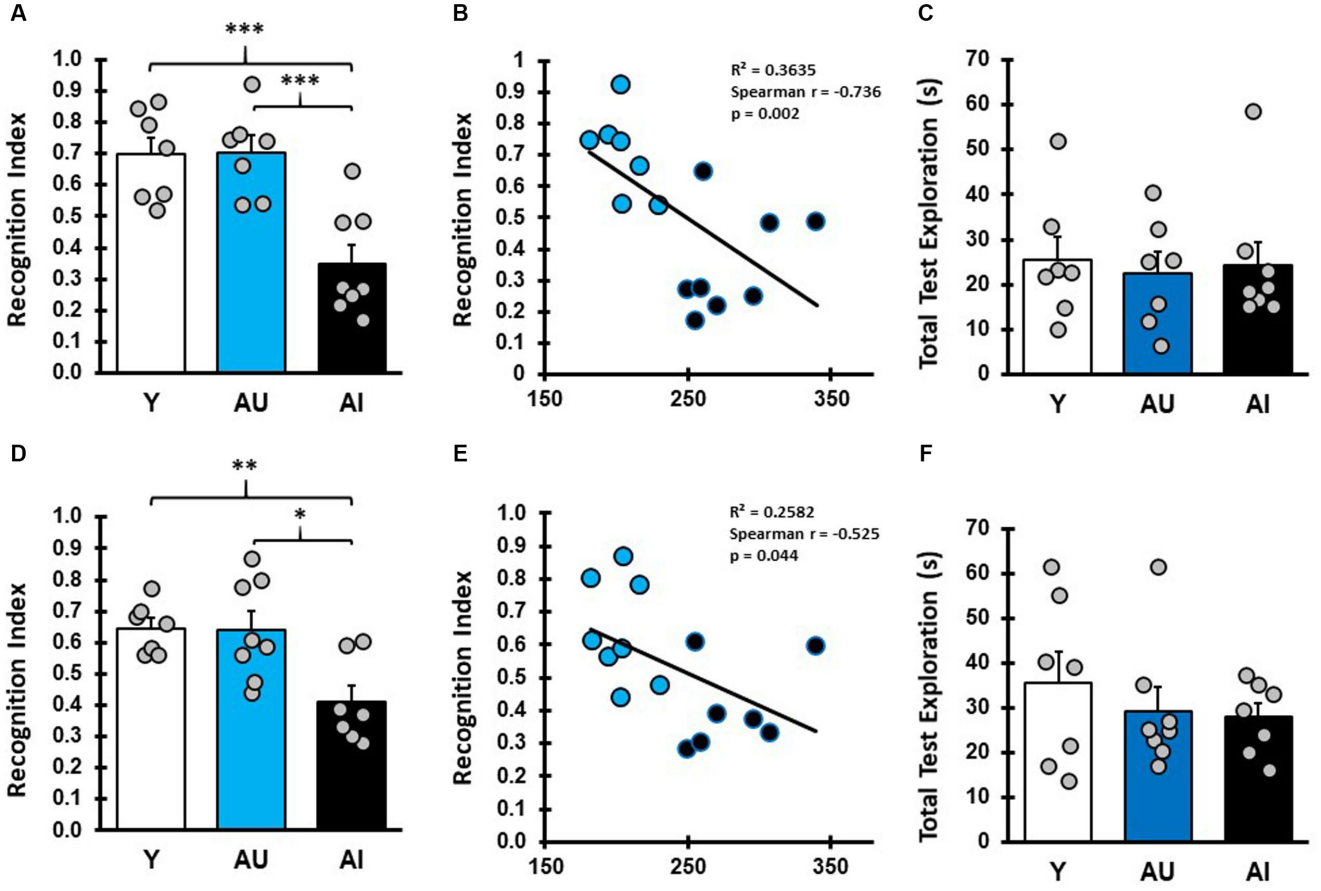
Aged rats with hippocampal dependent spatial learning deficits are impaired in long-term odor recognition memory. **(A)** Recognition index (RI) scores reflect the proportion of time spent exploring the novel odor during the test phase. RI scores for Y, AU, and AI animals on long-term odor recognition memory test 1 (LTM1) show that Y and AU animals perform similarly, while AI animals spent less time investigating the novel odor (Avg/SEM; Y: 0.697 ± 0.0545; AU: 0.703 ± 0.0508, AI: 0.350 ± 0.0591). **(B)** Correlation plots of LTM1 RI scores versus water maze LI scores in aged rats show that animals with higher learning index scores (worse water maze performance) displayed lower RI scores. **(C)** Total test phase odor vial exploration in LTM1 for Y, AU, and AI was not different across groups (Avg/SEM; Y: 25.51 ± 5.19, AU: 22.54 ± 4.51, AI: 24.37 ± 5.11). **(D)** Long-term memory test 2 (LTM2) RI scores for Y, AU, and AI phenocopy result from LTM1 with AI animals showing decreased investigation of the novel odor (Avg/SEM; Y: 0.65 ± 0.031, AU: 0.64 ± 0.06, AI: 0.41 ± 0.051). **(E)** Correlation plots of LTM2 RI scores versus water maze LI scores in aged rats. **(F)** Total test phase odor vial exploration in LTM2 for Y, AU, and AI was not different across groups (Avg/SEM; Y: 35.6 ± 7.08, AU: 29.29 ± 4.96, AI: 27.94 ± 3.06). ^***^ = *p* < 0.001, ^**^ = *p* < 0.01, ^*^ = *p* < 0.05.

To assess the relationship between hippocampal-dependent spatial learning performance and odor recognition memory, the characterized rats in this study were given a series of odor recognition memory tasks with varying delays and a Recognition Index (RI) score was calculated [(novel/novel + familiar]). Two long-term memory tasks were performed in which rats were tested 24 h after concluding the sample phase (Figure 2). In the first of these tests (LTM1, Figure 2A), Y and AU animals preferentially explored the novel odor vial more than expected by chance (Supplementary Table S1; one-sample *t* test, mean difference compared with 0.5: Y: *p* = 0.011, AU: *p* = 0.007). AI animals, however, preferentially explored the familiar odor more than chance (*p* = 0.0394), resulting in lower RI scores as a group, significantly differing from both Y and AU (One way ANOVA: *F* (2, 19) = 13.83, *p* = 0.0002, Tukey *post hoc*: Y vs. AU: *p* = 0.997; Y vs. AI: *p* = 0.0007; AU vs. AI: *p* = 0.0006), suggesting that they may have treated the familiar odor as if it were novel, as reported elsewhere (see Burke et al., 2010). This was not driven by a failure to sample the familiar odor during initial presentation, as total exploration time in the LTM1 sampling phase was similar across groups (*F* (2, 19) = 1.304, *p* = 0.295) and was not correlated with RI scores (Spearman, *r* (15) = 0.282, *p* = 0.31), and not due to a failure to investigate the odor vials during the test phase (Figure 2C; Supplementary Table S1; *F* (2, 19) = 0.08697, *p* = 0.917). To further examine the relationship between this impairment in long-term odor recognition memory and hippocampal-dependent memory in the aged animals, we plotted RI scores for LTM1 against LI scores for each animal (Figure 2B), identifying a significant negative correlation between the two test measures (Spearman, *r* (13) = −0.736, *p* = 0.0025).

### 3.2. Age-related odor recognition memory impairments are stable over repeated testing

To determine whether this impairment in odor recognition memory is stable over time for a given animal and across different odor pairs, animals were given a second Long-term Memory test (LTM2, Figure 2D) using different odors and in a different arena. Y and AU groups had above chance exploration of the novel odor, while AI performed at chance levels (Supplementary Table S1; one-sample *t* test, mean difference compared with 0.5: Y: *p* < 0.003, AU: *p* < 0.039, AI: *p* = 0.131). Again, RI scores for Y and AU rats were similar, while AI rats had significantly lower RI scores (Supplementary Table S1; one way ANOVA: *F* (2, 19) = 7.612, *p* = 0.0037, Tukey *post hoc*: Y vs. AU: *p* = 0.996; Y vs. AI: *p* = 0.0083; and AU vs. AI: *p* = 0.0079). Similar to LTM1, aged animals RI scores on LTM2 were negatively correlated with water maze LI scores (Figure 2E; Spearman, *r* (13) = −0.525, *p* = 0.0471). While total test phase odor vial exploration time in LTM2 was lower in the aged rats relative to Y (Figure 2F), there was not a statistical difference between the groups for sample phase exploration (Supplementary Table S1; one way ANOVA: *F* (2, 19):1.976, *p* = 0.1661) or test phase exploration (Figure 2F; Supplementary Table S1; one way ANOVA: *F* (2, 19) = 0.580, *p* = 0.5695) and sample phase exploration did not correlate with RI scores (Spearman, *r* (15) = 0.054, *p* = 0.853). Furthermore, there was a significant correlation between performance on LTM1 and LTM2 across all animals (Pearson, *r* (22) = 0.451, *p* = 0.040). Taken together, the results of LTM1 and LTM2 suggest that for aged animals, the ability to recall previously experienced odor cues after a long-term delay is a stable phenotype and that this impairment has a direct relationship with an individual’s MTL-dependent cognitive abilities.

### 3.3. Age-related spatial learning impairments do not predict short-term odor recognition memory impairments

**Figure 3:**
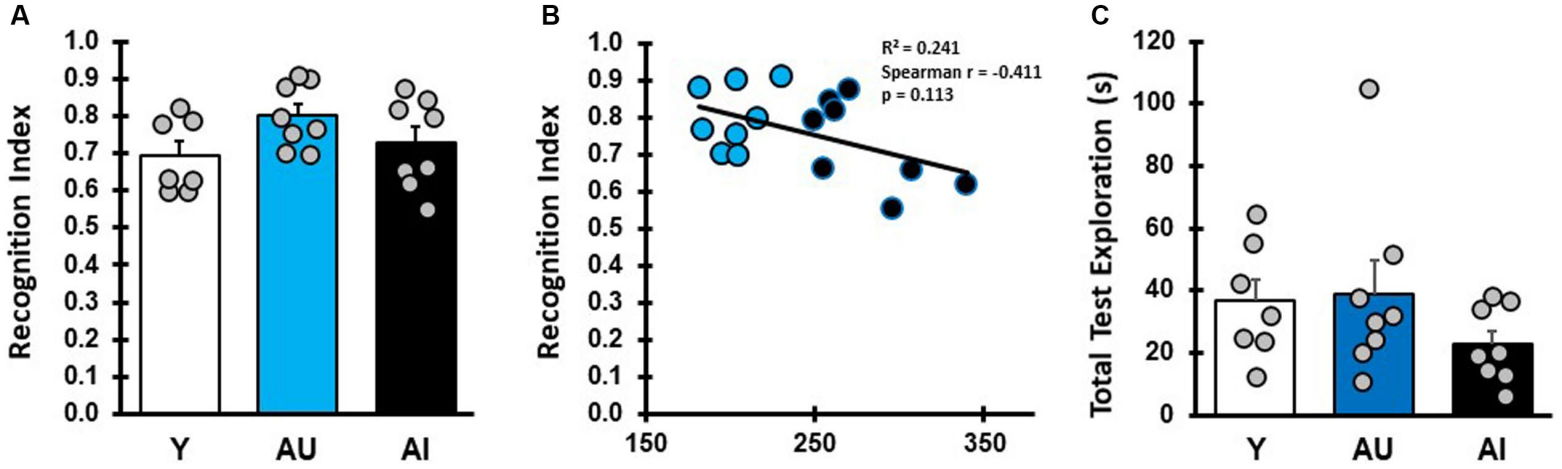
Short-term odor recognition memory test. **(A)** RI for Y, AU, and AI animals in a short-term memory test with a 5-min delay between sampling and test phases (Avg/SEM; Y: 0.694 ± 0.038, AU: 0.80 ± 0.03, AI: 0.73 ± 0.042). **(B)** Correlation between short-term memory RI scores and water maze LI scores in aged rats. **(C)** Total odor vial exploration during the short-term memory test phase (Avg/SEM; Y: 36.57 ± 7.0, AU: 39.09 ± 10.36, AI: 22.90 ± 4.26).

The failure of AI rats to explore the novel odor in these tests could result from an olfactory deficit or from a lack of novelty exploration preference. To determine whether this is the case, all rats were given a short-term memory test with a 5-min delay between sampling and testing phases (Figure 3). All young rats preferentially explored the novel odor vial presented in the test phase, demonstrating intact spontaneous novelty exploration under these conditions (Figure 3A). Similar to young adult animals, all aged animals preferentially explored the novel odor significantly more than chance in the test phase (Figure 3A; Supplementary Table S1; one-sample *t* test, mean difference compared with 0.5: Y: *p* = 0.0021, AU: *p* = 0.0001, AI: *p* = 0.001), with no statistical difference between groups (Supplementary Table S1; one way ANOVA: *F* (2, 20) = 2.13, *p* = 0.1446). Importantly, short-term memory RI scores were not correlated with MWM learning index scores (Figure 3B, Spearman, *r* (14) = −0.41, *p* = 0.113), indicating that the low RI scores for AI animals in the LTM tests was not due to an inability to identity the novel odor or a lack of innate novelty exploration preference. Aged rats as a group tended to have lower test phase exploration times relative to Y in the STM test (Figure 3C), but this was not significantly different (One way ANOVA: *F* (2, 20) = 1.319, *p* = 0.2896). The AU and AI, furthermore, did not differ in exploration during the sample phase (Supplementary Table S1; one way ANOVA: *F* (2, 20) = 4.372, *p* = 0.0266, Tukey’s *post hoc*: Y v AU: *p* = 0.3219, Y vs. AI: *p* = 0.0205, AU v AI: *p* = 0.2996) and sample phase exploration did not correlate with RI scores (Spearman, *r* (16) = −0.128, *p* = 0.635). These results demonstrate that under these experimental conditions, both AU and AI animals have intact odor recognition, can form a memory of a recently presented odor cue, and have intact novelty exploration preference after a short delay when the relevant cues are odorants.

## 4. Discussion

The current work was designed to determine the stability of within-subject, age-related performance across olfactory recognition memory assessments in rodents to serve as a behavioral assay for within-subject intervention studies. Young adult and aged Long-Evans rats were characterized for spatial learning ability via Morris water maze assessment, a well-established test of hippocampal function. As has been shown previously in this model (Gallagher et al., 1993), a subset of aged rats performed within the range of young adult animals while the rest performed outside this range, demonstrating impaired spatial learning. Rodent studies investigating the underlying neurological basis of this individual variability in age-related spatial learning have shown that Morris water maze performance is predictive of performance in other tests of spatial learning (Gallagher and Burwell, 1989; Colombo et al., 1997; Koh et al., 2010) as well as some tests of non-spatial learning (LaSarge et al., 2007; Robitsek et al., 2008).

The odor recognition memory test used here was based on spontaneous novel object (SOR) recognition paradigms which take advantage of rats’ preference for exploring novel stimuli (Ennaceur and Delacour, 1988). Two vials of the same odor were presented to young adult and aged rats, and after a short or long delay animals were assessed for their exploration of this familiar odor vial versus a novel one. Both young adult and aged animals displayed increased exploration of the novel odor vial following a short delay, indicating they formed a memory for the familiar odor and had intact odor discrimination and novelty exploration biases under these conditions. Following a long delay, aged rats had varying degrees of impairment in identifying the novel odor and the degree of this impairment paralleled that observed in the Morris water maze test for each aged rat. Furthermore, performance in a second test with a new pair of odors replicated these findings. Although RI scores for AI rats were slightly higher in the second long term memory test, individual performance was positively correlated across the two tests, suggesting that this impairment is replicable and generalizes across different odors.

It has been demonstrated that aged rodents are not impaired in their ability to recognize and preferentially explore novel objects when delays between sampling and testing are short (2–15 min) (Burke et al., 2010; Bergado et al., 2011; Arias-Cavieres et al., 2017) but are impaired relative to young adult animals with long-term delays (Cavoy and Delacour, 1993; Lukaszewska and Radulska, 1994; Pietá Dias et al., 2007; Burke et al., 2010; Aktoprak et al., 2013; Arias-Cavieres et al., 2017; Weiler et al., 2021), similar to animals with hippocampal inactivation (Hammond et al., 2004), perirhinal lesions (Ennaceur and Aggleton, 1997; Kesner et al., 2001), and aged human subjects (Davis et al., 2003). This suggests that the observed age-related memory impairment is due to memory load rather than impaired ability to perceive differences between objects.

SOR paradigms have been used to assess a variety of neurobiological domains in rats as well as in studies of cognitive aging and are particularly attractive for use with aging animals as they require relatively little training and habituation and do not require food deprivation. Similarly, exploration of odor cues in this paradigm provides a rich space of possible cue combinations and shares the advantages of SOR paradigms. In addition, our research program has made extensive use of olfactory cues in many complex behavioral paradigms in this rodent model (Schoenbaum et al., 2002; Robitsek et al., 2008) in an effort to characterize age-related changes in memory and cognition. Here we demonstrate further that odor memory impairments at 24-h delays parallel MTL-dependent spatial memory impairments and that these impairments are stable over repeated testing.

Alongside the current findings using assessments that depend on the MTL, there is evidence that aging occurs independently in different neurocognitive domains associated with distinct neural networks (as reviewed in Baxter and Gallagher, 1996; Gallagher and Rapp, 1997). For example, in a study of aged human subjects characterized for both MTL and frontal lobe functioning, Glisky et al. (1995) reported a lack of association between performance on MTL-dependent and frontal lobe-dependent tasks, suggesting these neurocognitive domains do not necessarily decline in parallel or at the same rate. Similarly, studies in rats have shown that aged rats exhibit individual differences in reversal learning and attentional set-shifting tasks that depend on the prefrontal cortex, but individual performance in those assessments was not systematically associated with Morris water maze spatial learning scores obtained in the same aged subjects (Barense et al., 2002; Schoenbaum et al., 2002). In that context, it is notable that aged rats in the current study showed close correspondence between MTL-dependent spatial learning abilities with repeated tests for recognition memory at long delays.

Simple within-subject assessments, such as the one tested here, can provide a basis for within-subject intervention studies to test for cognitive improvements in impaired subjects or to prevent decline from occurring. Further, it is intriguing to note that while cognitively unimpaired aged humans and AU rats phenocopy young adult subjects in behavioral cognitive tests, there is substantial evidence that aged unimpaired rats in this study population retain cognitive abilities by adaptive mechanisms rather than by maintaining a young-like brain state. For example, in this model, AU rats show enhanced recruitment of inhibitory mechanisms relative to both young adult and AI animals (Tran et al., 2018; Branch et al., 2019) and this inhibitory recruitment appears to be supportive of cognitive function (Koh et al., 2020). Indeed, the occurrence of hyperactivity in the hippocampus in human aging is also recognized as a prognostic indicator of further cognitive decline (Leal et al., 2017; Berron et al., 2019). Thus, additional work may make use of novel odor recognition tests to identify other neurobiological substrates of successful aging.

## Data availability statement

The raw data supporting the conclusions of this article will be made available by the authors, without undue reservation.

## Ethics statement

The animal study was approved by Johns Hopkins University Institutional Animal Care and Use Committee. The study was conducted in accordance with the local legislation and institutional requirements.

## Author contributions

AB and MG designed the work. LG performed the main experiments. AB wrote the manuscript with input from all co-authors. All authors contributed to the article and approved the submitted version.

## Supplementary material

### SUPPLEMENTARY FIGURE S1

Inter-rater reliability for odor exploration scoring. **(A)** Correlation and regression showing that the two raters (Raters 2 and 3) as compared to the experimenter (Rater 1) scored the behavior during the first long-term memory test, Pearson *r* = 0.96, *p* < 0.0001, Goodness of fit with Rater 2 (R2 = 0.94) and Rater 3 (R_2_ = 0.90). **(B)** Correlation and regression showing that the two raters (Raters 2 and 3) as compared to the experimenter (Rater 1) scored the behavior during the second long-term memory test, Pearson *r* = 0.89, *p* = 0.001, Goodness of fit with Rater 2 (R_2_ = 0.80) and Rater 3 (R_2_ = 0.82). **(C)** Correlation and regression showing that the two raters (Raters 2 and 3) as compared to the experimenter (Rater 1) scored the behavior during the short-term memory test, Pearson *r* = 0.78, *p* = 0.008, Goodness of fit with Rater 2 (R_2_ = 0.61) and Rater 3 (R_2_ = 0.51).

### SUPPLEMENTARY TABLE S1

Statistical tests and results. SEM: standard error of the mean, MD: mean difference, SS: sum of squares, df: degrees of freedom, MS: mean square, LL: lower limit, UL: upper limit, Test exp.: total test phase odor exploration, Sample exp.: total sample phase odor exploration.
