# Supplementary figures and images for: Individual differences in age-related neurocognitive outcomes: within-subject assessment of memory for odors
Source: Front Aging Neurosci. 2023 Sep 28;15:1238444. doi: 10.3389/fnagi.2023.1238444 (PMC10569039; doi:10.3389/fnagi.2023.1238444)

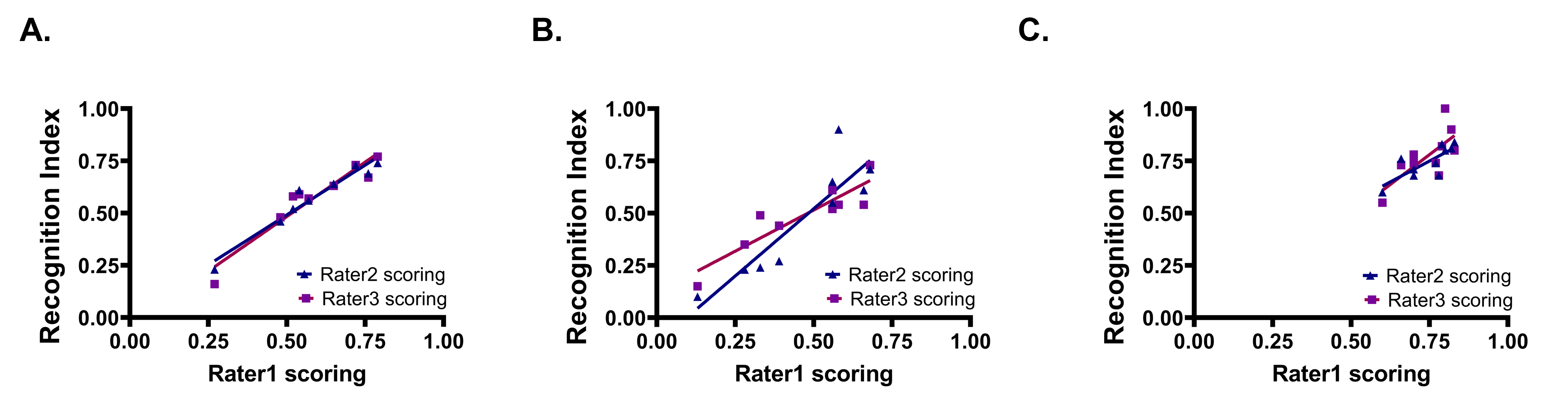

Supplement: Supplementary file 1 [file Image_1.TIF]

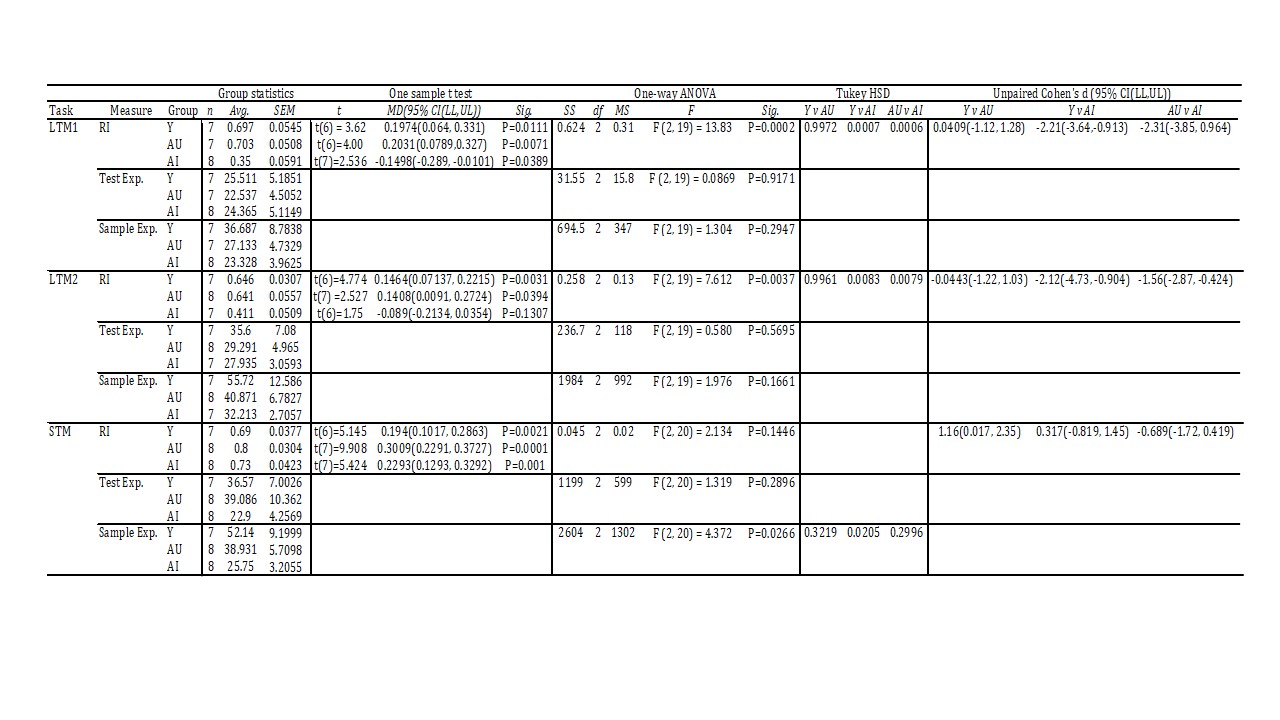

Supplement: Supplementary file 2 [file Image_2.JPEG]
